# Novel Prediction Method Applied to Wound Age Estimation: Developing a Stacking Ensemble Model to Improve Predictive Performance Based on Multi-mRNA

**DOI:** 10.3390/diagnostics13030395

**Published:** 2023-01-20

**Authors:** Lihong Dang, Jian Li, Xue Bai, Mingfeng Liu, Na Li, Kang Ren, Jie Cao, Qiuxiang Du, Junhong Sun

**Affiliations:** School of Forensic Medicine, Shanxi Medical University, 98 University Street, Yuci District, Jinzhong 030604, China

**Keywords:** wound age estimation, skeletal muscle contusion, stacking ensemble learning, multiple mRNAs, forensic science

## Abstract

(1) Background: Accurate diagnosis of wound age is crucial for investigating violent cases in forensic practice. However, effective biomarkers and forecast methods are lacking. (2) Methods: Samples were collected from rats divided randomly into control and contusion groups at 0, 4, 8, 12, 16, 20, and 24 h post-injury. The characteristics of concern were nine mRNA expression levels. Internal validation data were used to train different machine learning algorithms, namely random forest (RF), support vector machine (SVM), multilayer perceptron (MLP), gradient boosting (GB), and stochastic gradient descent (SGD), to predict wound age. These models were considered the base learners, which were then applied to developing 26 stacking ensemble models combining two, three, four, or five base learners. The best-performing stacking model and base learner were evaluated through external validation data. (3) Results: The best results were obtained using a stacking model of RF + SVM + MLP (accuracy = 92.85%, area under the receiver operating characteristic curve (AUROC) = 0.93, root-mean-square-error (RMSE) = 1.06 h). The wound age prediction performance of the stacking models was also confirmed for another independent dataset. (4) Conclusions: We illustrate that machine learning techniques, especially ensemble algorithms, have a high potential to be used to predict wound age. According to the results, the strategy can be applied to other types of forensic forecasts.

## 1. Introduction

Accurately estimating wound age is one of the central issues in daily forensic practice [1]. Wound age refers to the time an individual survives after an injury is inflicted. It provides clues for criminal investigations to confirm the occurrence and development of violent incidents and delineate possible criminal suspects [2]. Many studies have shown that the pattern of characteristic biomolecular changes after an organismal injury can be applied to wound age prediction [3,4]. Although some biomarkers have shown great promise in wound age inference, applications are still very limited [5,6,7,8].

Previous studies argued that mRNA molecules, which serve as direct or indirect templates for other molecular markers, are expressed regularly with wound age [9]. Encouraging results have also been recently obtained in multi-mRNA-based wound age estimation [10,11,12]. Also, since expression changes at the mRNA level are relatively earlier than at the protein level, it demonstrates excellent value in early wound age inference [13,14]. Although mRNA is less stable than protein, it has been detected in a long-preserved sample [15,16]. Therefore, other independent investigations are needed before using mRNA for wound age estimation in daily practice.

In this context, how can multi-mRNAs’ characteristics accurately converge to the objective and quantified injury time? Machine learning methods provide a choice that has recently gained momentum in forensic science and have the potential to overcome the lack of current methods [17,18,19]. Research on machine learning models for wound age prediction based on the changing pattern of mRNA markers is imperative. However, machine learning applications in wound age estimation have rarely been reported.

Surprisingly, medical studies have obtained accurate prediction results using ensemble learning strategies, which can combine different base algorithms to solve the same problem [20,21,22]. According to Shaw et al. [23], ensemble learning significantly improved over any single model in performance. Additionally, a few studies have further indicated that stacking has the most powerful prediction ability for complex issues among the most common ensemble learning methods of stacking, boosting, and bagging [24,25,26]. Notably, a recent study reveals that base learners of the ensemble stack could be “good but different” [27].

Therefore, we explore the potential of machine learning algorithms for wound age estimation based on nine mRNA expression characteristics. The prediction performances of stacking ensembles combining different base algorithms were compared, focusing on the stacking ensemble that exhibits the highest prediction power.

## 2. Materials and Methods

### 2.1. Ethics

This study was conducted in compliance with the ARRIVE guidelines and evaluated and approved by the Institutional Animal Care and Use Committee of Shanxi Medical University of China with approval number 2016LL151. Animals received humane care under the Guide for the Care and Use of Laboratory Animals of the Ministry of the People’s Republic of China.

### 2.2. Animals

Sprague–Dawley rats (6–8 weeks, male, pathogen-free) were obtained from the Experimental Animal Center (Shanxi, China). Rats were group-housed (2–3 animals per cage) under a 12-h light–dark cycle at 22–24 °C and 40–60% relative humidity in individually ventilated cages and had ad libitum access to food and water. The 56 animals were randomly assigned into a control group and six contused groups (*n* = 8/group). The injury group site was swabbed at 4-, 8-, 12-, 16-, 20- and 24 h after injury. The control group is defined as 0 h. Another 14 rats as external validation were randomly allocated to control and contusion groups (*n* = 2/group).

### 2.3. Skeletal Muscle Contusion and Sample Collection

As described previously, skeletal muscle contusion was performed on the rats [28]. Briefly, the skeletal muscle wounds were created on the right posterior limb using a 500 g weight that fell freely from 30 cm after anesthesia. The anesthesia was an intraperitoneal injection of pentobarbital (40 mg/kg). After wounding, each rat was housed in a sterilized cage and given enough food and water.

The samples, weighing about 100 mg, were put into liquid nitrogen, then stored at −80°C. In the same way, the samples of the control groups were obtained without injury.

### 2.4. Relative Quantitative Protocol of Nine mRNAs’ Expression

Total RNA was extracted from the skeletal muscle tissues using RNAiso Plus 9108 (Takara Bio, Shiga, Japan). The concentration (ng/mL) and purity of the freshly extracted total RNA were measured using a microplate reader (Infinite M200 Pro; TECAN, Zurich, Switzerland). The total RNA integrity was measured using the Agilent RNA 6000 Nano kit and Agilent 2100 (Agilent Technologies, Palo Alto, CA, USA). RNAs were reverse-transcribed into cDNA using a Prime Script TM RT Master Mix kit (Takara Bio).

The primers and probes were designed for nine mRNA using the Allele ID 6 software (Premier Biosoft International, Palo Alto, CA, USA) and synthesized by Sangon Biotech (Shanghai, China; Supplementary Material S1, Table S1). According to the Premix Ex Taq ™ kit (Takara Biotechnology Co., Ltd., Dalian, China), configuration reaction mixture, we used Bio-Rad CFX384 fluorescence quantitative PCR system (Hercules, CA, USA) for real-time quantitative PCR (RT-qPCR).

The RT-qPCR was performed in a 25 μL reaction system using the Premix Ex Taq Kit (Takara Biotechnology Co., Ltd., Dalian, China) [29]. The contents of the amplification mix and the thermal cycling conditions were set according to the instructions. The amplification of each mixture contained four mRNA primers and probes, including two reference genes and two target genes. The RT-qPCR procedure was repeated three times for each sample. Negative controls were monitored simultaneously during each run. Relative expression levels of the mRNAs were computed using the statistical model (1 + Eff.) – ΔΔCt, normalized with the geometric mean of the reference gene (RPL13 and RPL32 mRNAs) levels [30], where ΔCt = ΔCt(target gene) – ΔCt (reference gene) and ΔCt = Ct(for each time point) – Ct(for control).

### 2.5. Model Development and Validation

**Figure 1:**
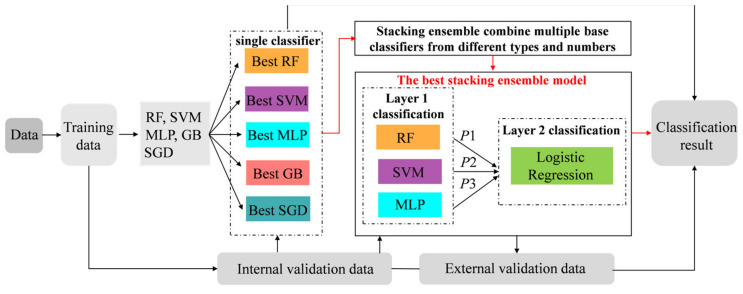
The flow chart of model training and validation.

Predictive models were built using internal validation datasets such as Random Forest (RF), Support Vector Machine (SVM), Multilayer Perceptron (MLP), Gradient Boosting (GB), Stochastic Gradient Descent (SGD), and Stacking Ensemble learning. They were implemented based on scikit-learn in Python. Figure 1 shows three parts of the development and validation of models:

(i) Selection of the optimal parameter of the base classifiers: The hyperparameters (RF, SVM, MLP, GB, SGD) were optimized within each fold by creating a five-fold training set according to grid search. The learners were then retrained using optimal hyperparameters on the internal validation data to determine the best base models.

(ii) Construction of stacking ensemble models: We developed a two-level stacking ensemble model consisting of multiple basic classifiers and a single meta-learner. We employed the trained learners (RF, SVM, MLP, GB, SGD) for the base classifiers. The logistic regression (LR) as a meta-classifier was used to learn the truth from the base learners’ predicted scores (P1, P2, P3). The 26 stacking ensemble models were developed: combining two base learners (RF + SVM, RF + MLP, RF + GB, RF + SGD, SVM + MLP, SVM + GB, SVM + SGD, MLP + GB, MLP + SGD, SGD + GB); combining three base learners (RF + SVM + MLP, RF + SVM + GB, RF + SVM + SGD, RF + MLP + GB, RF + MLP + SGD, RF + GB + SGD, SVM + MLP + GB, SVM + MLP + SGD, MLP + GB + SGD); combining four base learners (RF + SVM + MLP + GB, RF + SVM + MLP + SGD, SVM + MLP + GB + SGD, RF + MLP + GB + SGD, RF + SVM + GB + SGD); ensemble five base learners (RF + SVM + MLP + GB + SGD). The best-performing stacking ensemble models were acquired.

(iii) Data splits for training and validating: The data were randomly divided into 80% as a training set and 20% as an internal validation set. New data generated on another 14 rats were used as external validation to confirm the effectiveness of the best-performing stacking model and base learner.

### 2.6. Evaluation of the Predictive Performance

To evaluate the predictive performance of each model, the generalized area under the receiver operating characteristic curve (AUROC), accuracy (ACC), precision, recall rate, F1 score, and root-mean-square-error (RMSE) were utilized [31]:

(1) $$\mathrm{Accuracy}=\;\frac{TP+TN}{TP+TN+FP+TN},$$

(2) $$\mathrm{Recall}\;\mathrm{rate}=\frac{TP}{TP+FN},$$

(3) $$\mathrm{Precision}=\frac{TP}{TP+FP},$$

(4) $$F1-\mathrm{score}=\frac{2Precision\times Recall}{Precision+Recall},$$

(5) $$\mathrm{RMSE}=\sqrt{\frac{1}{m}\sum_{i=1}^{m}{\left(y_{i}-\bar{y_{i}}\right)}^{2}},$$

where: *TP*: true positive, *TN*: true negative, *FP*: false positive, *FN*: false negative. The generalized AUROC is plotted with the *TP* rate against the *FP* rate for the multiclass classification. RMSE: *m*: Number of training data, *i*: the i-th training data, *y_i_*: actual value. $\bar{yi}$: prediction value.

RMSE is used to quantify the model’s error in predicting the time to injury. To calculate the overall performance rank of the 26 stacking ensemble models, the Borda count was used to summarize each of the five performance indicators. The Borda count method is as follows [32]: in an election of N candidates, give 1 point for the last place, 2 points for the second from last place, and so on to the top of the ballot. A first-place vote is worth N points. For example, for 3 voters, with 1st choice ABC, 2nd choice CBA, and 3rd choice BCA, A gets 5 points, B gets 7 points, and C gets 6 points. Therefore, B is the winner. This study used the above five evaluation indicators as Borda count voters.

Confusion matrices are frequently used in the field of machine learning to contrast classification results with actual measured values. The ordinate of the confusion matrix represents the predicted value, and the abscissa of the matrix is the actual value of the models. In the confusion matrix, samples that occurred on the diagonal showed that the model predicted accurately, whereas those outside of the diagonal represented incorrect samples.

## 3. Results

### 3.1. The Characteristics of Different Genes in Contused Skeletal Muscle

**Figure 2:**
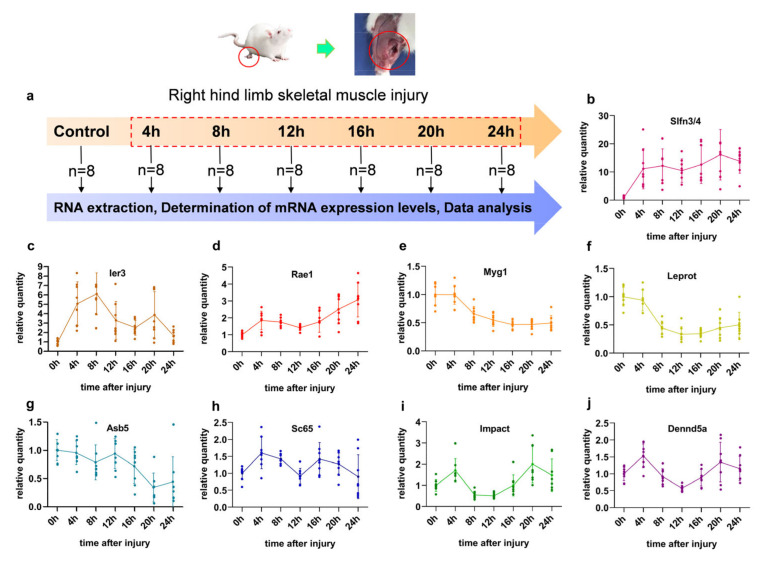
Sampling of skeletal muscle contusion of rats and detection of gene expression profiles. (**a**) The contused skeletal muscle was collected from rats at different time points after injury. (**b**–**j**) The expression profiles of nine mRNAs. Each dot represents a sample. The 0 h in the graph represents the control group.

After skeletal muscle injury, the sampling was obtained at seven time points (Figure 2a). The expression of the nine target mRNAs was determined using RT-qPCR throughout the post-traumatic period (Figure 2b–j, Supplementary Material S1, Table S1). The injury caused a statistically significant expression change in Slfn3/4, Ier3 (expect 16- and 24 h), and Rael (expect 12 h) mRNAs at 4, 8, 12, 16, and 20 h post-injury compared to the control group (*p* < 0.05) (Figure 2 b–d), while the expressions of the other genes changed after either 8, 12, 16, 20, and 24 h (Myg1, Leprot) or 20 and 24 h (Asb5) (*p* < 0.05) (Figure 2d–f). Furthermore, the expression of Sc65 changed statistically significantly at 4, 8, and 16 h, but Impact occurred at 4 h and 20 h (*p* < 0.05) compared to the control group. After 4 and 20 h after injury, Dennd5a’s expression level was higher than that of the control group (0 h); however, at 12 h after injury, it was lower than that of the control group (0 h) (*p* < 0.05) (Figure 2j).

### 3.2. Performance of the Five Basic Classifiers for Wound Age Estimation

**Figure 3:**
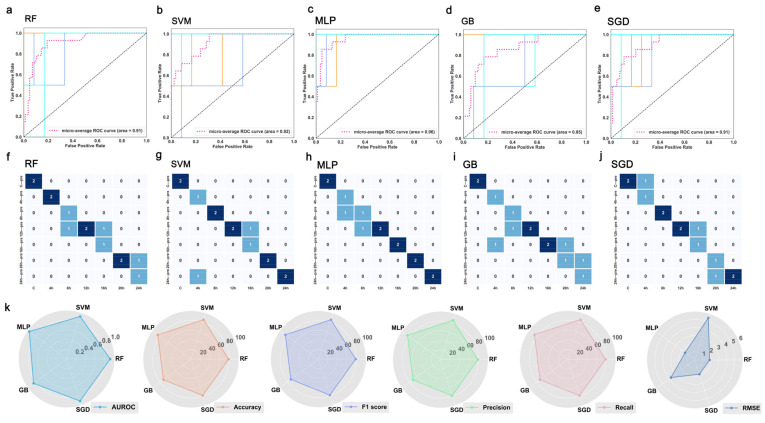
Predicted wound age against actual wound age for SVM, RF, MLP, GB, and SGD on the internal validation. The AUROC (**a**–**e**) and the confusion matrix (**f**–**j**) of five basic classifiers. The horizontal axis is the actual value, the vertical axis is the predicted value, and the diagonal line indicates that the prediction is correct. (**k**) Performance of five classifies on the internal validation. Abbreviations: RF: random forest; SVM: support vector machine; MLP: multilayer perceptron; GB: gradient boosting; SGD: stochastic gradient descent; AUROC: area under the receiver operating characteristic curve.

We obtained the optimal parameter combinations of the base learners using the grid search strategy (Supplementary Materials S2). The results of testing the base classifiers with the optimal combination of parameters using the internal validation dataset are shown in Figure 3. In terms of the overall area under the receiver operating characteristic curve (AUROC), the five classification models have adequate prediction power (above 0.85) for wound age estimation. Remarkably, the random forest (RF), support vector machine (SVM), and multilayer perceptron (MLP) achieve good predictions with corresponding AUROCs of 0.91, 0.92, and 0.96, respectively (Figure 3a–c). The confusion matrix further shows that the MLP has excellent predictive power (Figure 3f–j).

The calculation results in Figure 3k show that the accuracy, F1 score, precision, and recall rate exhibit the same values, and thus the performances were evaluated in terms of accuracy. Obviously, MLP and SVM outperform RF, stochastic gradient descent (SGD), and gradient boosting (GB), achieving an accuracy of 85.71%. It is worth mentioning that the root-mean-square-error (RMSE) for the MLP model (1.51) was 3.94 h below that of the SVM (5.45). Therefore, MLP may be the best for wound age estimation (Figure 3). As shown in Figure 3, the performances of RF and SGD are fairly similar (accuracy =78.57%, AUROC = 0.91, RMSE = 1.85 h), but the former was more sensitive to the 0 h. Note that the GB model has the worst performance among the five algorithms.

### 3.3. Comparison of Prediction Power of Multiple Stacking Ensembles

**Figure 4:**
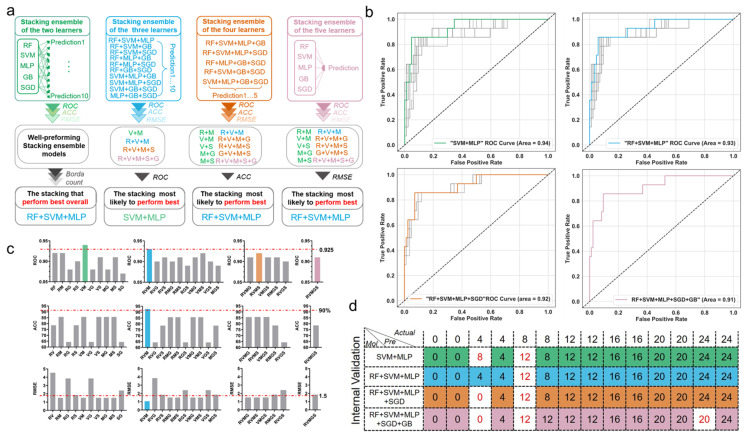
The comparison of 26 stacking models. (**a**) The workflow of the assessment of the stacking ensembles. (**b**) The AUROC of stacking models. The top left panel represents the 10 stacking models combing the two basic models with the well-performing model indicated with a colored line. Similarly, the top right, bottom left, and bottom right represent stacking models combing three, four, and five basic models, respectively. (**c**) Performances of 26 stacking models in terms of AUROC, accuracy, and RMSE. The horizontal coordinate represents different stacking models combing different basic learners. (**d**) Confusion matrix of best-performance stacking models. Abbreviations: RF: random forest; SVM: support vector machine; MLP: multilayer perceptron; GB: gradient boosting; SGD: stochastic gradient descent; “RF + SVM + MLP”: stacking model based on RF, SVM, and MLP; AUROC: area under the receiver operating characteristic curve; RMSE: root-mean-square-error.

Stacking ensemble strategies can combine multiple ML algorithms to enhance the predictive power of a single algorithm. To explore the predictive power of stacking models when combining different base classifiers, we compared the performances of 26 stacking models developed by combining two (ten in total) or three (ten in total) or four (five in total), or five (one in total) basic classifiers on the internal validation sets (Figure 4a). The calculation results show that the accuracy, precision, recall rate, and F1 scores exhibit the same values, and thus the performances were evaluated in terms of AUROC, accuracy, and RMSE.

We first evaluated the stacking models by combing the same quantities of basic models individually using AUROC. As shown in Figure 4b, the stacking ensemble of “SVM + MLP” is well-performing, with the greatest AUROC of 0.94 among the stacking ensembles combining two basic classifiers. Its performance is better than that of the stacking ensembles of “RF + SVM + MLP” or “RF + SVM + MLP + SGD”, although their AUROC was the highest among the stacking ensembles combing three or four basic models.

Although the stacking combination of “SVM + MLP” outperforms the other models in terms of AUROC, the stacking model of “RF + SVM + MLP” is optimal in terms of accuracy and RMSE, with an accuracy of 92.85% and RMSE of 1.06 h (Figure 4c). Further, Borda counts were used to compare the overall performances of the different stacking models. The stacking ensemble of “RF + SVM + MLP” outperforms the other stacking ensemble algorithms. The confusion matrix further shows that only one sample was incorrectly classified as 12 h instead of 8 h for the stacking model “RF + SVM + MLP”, indicating it may be the best choice for wound age estimation (Figure 4d).

**Figure 5:**
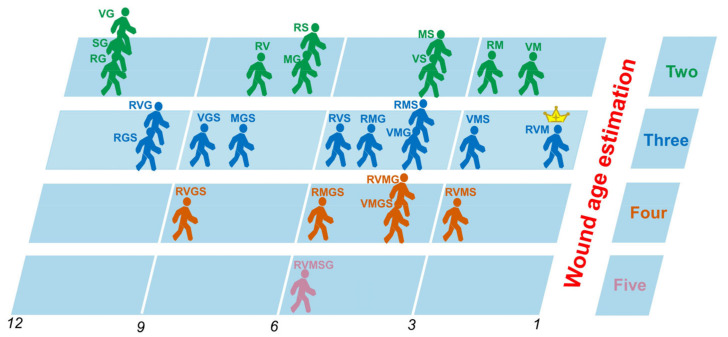
Overall performance ranking of the 26 stacked ensemble models based on the Borda count. Abbreviations: R: random forest; V: support vector machine; M: multilayer perceptron; G: gradient boosting; S: stochastic gradient descent; “R + V + M”: a stacking model based on RF, SVM, and MLP.

In addition, we compared all the stacking ensemble models (26 in total) according to the Borda count. As shown in Figure 5, the stacking ensemble of “RF + SVM + MLP” outperforms the other stacking ensemble algorithms. In summary, the overall performances of the 26 stacking ensembles can be ranked from the best to the worst as follows: R + V + M > V + M > R + M ≈ V + M + S ≈ R + V + M + S > M + S ≈ V + S≈V + M + G≈R + M + S≈V + M + G + S ≈ R + V + M + G> > M + G ≈ R + M + G > R + M + G + S ≈ R + V + M + G + S≈R + V + S > M + G ≈ R + S > M + G + S≈R + V > R + V + G + S > V + G + S > R + S + G > R + V + G > S + G≈ R + G≈V + G.

### 3.4. Further Comparison of the Performance of the Best-Performing Stacking Model and the Basic Classifiers

**Figure 6:**
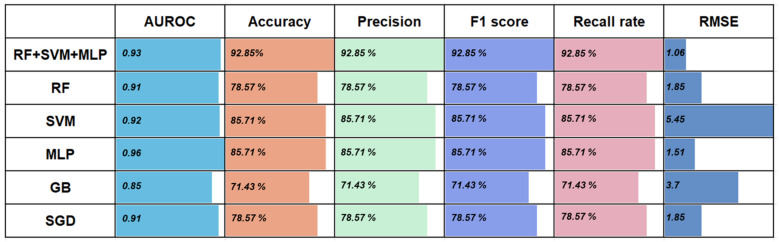
The performance of the best-performing stacking ensemble model and five base models. Abbreviations: RF: random forest; SVM: support vector machine; MLP: multilayer perceptron; GB: gradient boosting; SGD: stochastic gradient descent; AUROC: area under the receiver operating characteristic curve; RMSE: root-mean-square-error.

Figure 6 shows that the stacking model of “RF + SVM + MLP” performed better than all the basic classifiers on the internal validation set for wound age estimation; in particular, compared to GB, RF or SGD, and RF or SVM, the prediction accuracy was improved by 21.42%, 14.28%, and 7.14%, respectively. The smaller the RMSE is, the better the model’s performance is. The best-performing stacking model has the smallest RMES, followed by MLP. Unfortunately, the AUROC of the stacking model (RF + SVM + MLP) is decreased by 0.3 compared to the highest AUROC of the base learners (MLP).

### 3.5. Validation for the Best-Performing Stacking Ensemble and the Optimal Base Classifier

**Figure 7:**
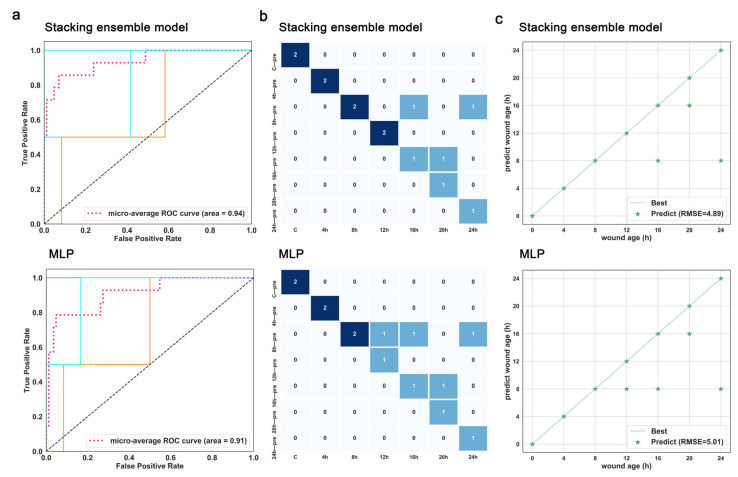
Comparison of the prediction performance between the stacking model of “RF + SVM + MLP” and MLP for wound age estimation in external validation. (**a**) AUROC, (**b**) confusion matrix, (**c**) RMSE of the stacking model (RF + SVM + MLP) and MLP. The pattern on the diagonal line indicates that the estimated value matches the actual value. The greater the error, the greater the distance between scatter and line. Abbreviations: RF: random forest; SVM: support vector machine; MLP: multilayer perceptron; GB: gradient boosting; SGD: stochastic gradient descent; AUROC: area under the receiver operating characteristic curve; RMSE: root-mean-square-error.

The promising stacking model of “RF + SVM + MLP” and MLP were tested separately using an external validation set from another 14 rats. Figure 7 demonstrates that the best-performing stacking model also slightly outperforms MLP and achieves an AUROC of 0.94, an accuracy of 78.57%, and an RMSE of 4.89 h. However, its performance is lower than on the internal verification data and still has room for improvement. Overall, the developed stacking model based on RF, SVM, and MLP seems preferable for estimating wound age.

## 4. Discussion

Skeletal muscle tissue is often used to infer the age of an injury [1,33]. The time-dependent expression of mRNA allows a reliable estimation of wound age after skeletal muscle injury [5,7,34]. The multi-mRNA combination provides an excellent subject for applying forensic wound age diagnostics, particularly for determining the age of 0–24 h wounds [35]. Recently, the advent of machine learning based on molecule biomarkers has facilitated new applications for the evaluation of wound age. For this reason, we developed a stacking ensemble model based on the expression characteristics of nine mRNAs for wound age prediction.

In this paper, we propose a multi-mRNA biomarker-based estimation system by applying an integrated strategy, which may be an accurate alternative to that can significantly change the current forensic injury time diagnosis pathways and decision-making methods. This approach is powerful as it blends a heterogeneous group of algorithms that expose distinct yet complementary aspects of the data. Furthermore, the Python computational environment enables transportable model development based on its standardized data structure and external validation dataset. Our model has portability and reproducibility, overcoming the limited usefulness in practice due to disclosing the model code in this study.

Researchers have attempted to combine multiple indicators to predict wound age, but few have discussed how to improve the accuracy and precision of their predictions with mathematical models [3]. The current study found that reasonable interval accuracy is often only achieved at the expense of precision. Our results of the stacking ensemble model (accuracy = 92.85%, RMSE = 1.06 h, AUROC = 0.93) achieved the highest interval accuracy and precision within 24 h. In Barington et al.’s study [36], the principal component analysis was developed with high precision (about ±2 h), but only if they divided the bruises within ten hours into three age intervals for wound age prediction.

In our previous studies, a Fisher discriminant analysis was constructed with a prediction RMSE of 11 h for wound age within 48 h [12]. The stacking model is higher than previously produced by multivariate statistical analysis for the wound age prediction (RMSE = 1.06 h for the internal set, RMSE = 4.89 h for the external group). Notably, this study used fewer genes (nine) than the previous (14 genes). Additionally, Fisher discriminant analysis was performed again based on the spatial distribution of neutrophils, and 100.0% and 95.0% of the original and cross-validated cases were correctly classified. The above studies further show that machine learning methods produce accurate results for age estimation 0–24 h after injury when using meaningful biomarkers strongly related to wound age changes.

The external validation results suggest that our model could be applied to data other than the development data, indicating that our model may be transportable and applicable. However, the prediction power of the stacking ensemble model showed a relatively lower external prediction power than that of the internal. This might be because of individual differences in the gene expression data among rats since different batches of rats were used at different times. Therefore, large sample sizes are necessary for accurate modeling in the future.

Generally, a combination of highly sensitive yet specific biomarkers is important for wound age estimation. Functionally, in this study, mRNAs are related to the process of inflammation and repair [12]. In particular, Asb5, Myg1 [37], and Sc65 [38] are involved in the activation and differentiation of satellite cells. Rae1 is closely related to the energy supply [39]. Ier3 [40], Leprot [41], Impact [42], and Slfn3/4 [43] play a significant role in the regulation of inflammation and the immune system. Dennd5a indirectly promotes autophagy by activating Rab39 [44]. Using multi-mRNAs of time-dependent participation in phases of skeletal muscle regeneration, we could indirectly understand how the ensemble model predicted wound age.

Several authors have observed that the model robustness improves when integrating machine learning algorithms with different nonlinear prediction abilities, learning abilities, and fault tolerances [45,46]. In this study, the stacking model of “Random Forest (RF) + Support Vector Machine (SVM) + Multilayer Perceptron (MLP)” achieves a great RMSE of 1.06 h, even though SVM, as one of the basic learners, has the largest RMSE of 5.45 (Figure 6). Additionally, the average RMSE is reduced by 1.88 h compared to the base model. These improvements in predictive ability can be explained by the fact that stacking ensemble learning exploits the best prediction from each algorithm [47].

We also note unexpected results about the stackings of different quantity and variety base models. According to Wolpert (1992) [48], an optimal combination of heterogeneous base learners provides the highest predictive power in a stacking ensemble. In this study, the basic model RF and Stochastic Gradient Descent (SGD) have the same predictive power on the internal validation data (accuracy of 78.57%, RMSE of 1.85 h, AUROC of 0.91), but the performance of the “SGD + SVM + MLP” ensemble is worse than that of the “RF + SVM + MLP”, which highlights the advantage of RF over SGD algorithms on our data. One possible reason is that the SGD algorithm is considered an optimization method for linear classifiers such as the SVM [49]. Wu et al. [50] confirmed the superior performance of combining algorithms from different categories over combining algorithms from the same category. It should be noted that the ensembles of Gradient Boosting (GB) and other algorithms, such as the “RF + SVM + MLP + GB” combination, also show weak predictive abilities. One possible reason is that GB does not fit small-sample-size data (Figure 3).

Our study faced several limitations. First, our analysis was performed on rats. Simple animal models can provide highly reproducible working and homogenous results but often do not reflect the human situation. Hence, human samples are required to verify the validity and accuracy of the predictive stacking model in the future. Moreover, given the limitations of only seven time points, we will set up more experiment points to optimize the targeted machine learning algorithm for wound age estimation in the future.

In forensic research, our stacking ensemble model provides a strategy for integrated multi-molecular prediction. This statement may be applicable to future studies from a methodological standpoint. On the one hand, the stacking method can be used to solve a variety of multilevel classification problems in forensics, such as identifying wounds sustained a long time before a person’s death. Furthermore, because the model is multiclass, it can easily be expanded by changing or adding basic learners for each class that correspond to the desired expected outcome (e.g., postmortem interval and age prediction). Furthermore, from an economic standpoint, we present replication-friendly model run scripts in this study. We will further test and encapsulate these programs in the future, making forecasts implementable even by novices.

## 5. Conclusions

The application of integrated strategies to construct machine learning models based on validated mRNA markers enables objective and accurate wound age assessment. We tested 26 stacking models and five base classifiers and discovered that superimposed ensemble models based on RF, SVM, and MLP classifiers have a higher predictive power on both internal and external validation data, implying that stacking ensemble models have the potential to significantly improve forensic wound age estimation. We demonstrate the feasibility of machine learning when multiple biomarkers are combined, which is required before introducing larger training sample sizes and extensive human data studies.

## Data Availability

The original contributions presented in the study are included in the article/Supplementary Material. Further inquiries can be directed to the corresponding authors.

## Supplementary Materials

The following supporting information can be downloaded at: https://www.mdpi.com/article/10.3390/diagnostics13030395/s1, Supplementary Material S1: Primers and probes used for RT-qPCR; Supplementary Material S2: Five optimal hyperparameter combinations of machine learning models; Supplementary Material S3: Python code for models of training and validation.
