# Supplementary material for: Novel Prediction Method Applied to Wound Age Estimation: Developing a Stacking Ensemble Model to Improve Predictive Performance Based on Multi-mRNA
Source: Diagnostics (Basel). 2023 Jan 20;13(3):395. doi: 10.3390/diagnostics13030395 (PMC9914838; doi:10.3390/diagnostics13030395)
Supplement: Supplementary file 1 [file diagnostics-13-00395-s001.zip › Supplementary_Material S1.pdf]

Table S1 Primers and probes used for RT-qPCR

Gene symbol	GenBank accession no.	Nucleotide sequence	Size of products (bp)	Effici -ency (%)
RPL13	NM_031101.1	F5'-TCGTGAGGTGCCCTACAGTTAG-3'<br>P5'-CACACCAAGGTCCGGGCTGGCAG-3'<br>R5'-GGTGCGTGCCATTTTCTTGTG-3'	107	100.4
RPL32	NM_013226.2	F5'-ATCTGGCCCTTGAATCTTCTCC-3'<br>P5'-TGTCGATGCCTCTGGGTTTCCGCC-3'<br>R5'-AGAGGACCAAGAAGTTCATCAGG-3'	115	105.2
Rae1	NM_001033708	F5'-AAGCTGAAGACCTCAGAGCAG-3'<br>P5'-CCGTGGCAGCGTGTGGCTTCAACC-3'<br>R5'-TTATAAAACTCATGCCCCTTGGAC-3'	119	97.9
Ier3	NM_212505	F5'-CGTGCGTCCGAACACTTCTC-3'<br>P5'-CGAAAACGCAGCCGACGGGTGCTC-3'<br>R5'- AATGTTGGGTTCCTCGGTTGG-3'	100	100.4
Leprot	NM_020099	F5'-GGGATTGTTGTTTCTGCCTTTGG-3'<br>P5'-TGCCAGCCAGCACAAGACCACAGG-3'<br>R5'-GCCTTGGATCGTGAGGAAAATAAC-3'	120	100.0
Impact	NM_001012235	F5'-AAGGTTCTTGCCAAGTTGTATGAG-3'<br>P5'-TCGCCAGTGCCACCCACAACATCT-3'<br>R5'-GCTGTTTCTCCATCATCTTCGG-3'	128	99.7
Asb5	NM_001044247	F5'-GGTCGTCTTCTTGCTCTGAGG-3'<br>P5'-CCACATGGTCACCCAGGCAGGCTT-3'<br>R5'-TCCAGCTTCCAGGAGAGTCC-3'	138	99.8
Sc65	NM_021581	F5'-GGAGATGAGTCCCTCACTGATC-3'<br>P5'-CCGCTCCATGTGTTCTGTGCTGCT-3'<br>R5'-AGCAAAGACGGTCATATAATCAGC-3'	144	99.5
Myg1	NM_001005545	F5'-ACCTCGCAACAACCTCATGG-3'<br>P5'-CGAATCGGGACGCACAACGGCAC-3'<br>R5'-CCGAGTCCGCACAATCTCTG-3'	133	93.7
Dennd 5a	NM_001107546	F5'-TACCATCCGTCAGCCCAAAC-3'<br>P5'-CCTGTCTCCCTCGGTCATTGCCCA-3'<br>R5'-CCCATCTTCTCTACCAGCATCC-3'	129	100.3
Slfn3/4	NM_053687	F5'-AAAGGCCCTCTTCAGTCAAGC-3'<br>P5'-CTGCCACACAGTCCCCGTAGCTGC-3'<br>R5'-TGAGAACAGTTTCCCGCAGAG-3'	150	104.4
