# Supplementary material for: Novel Prediction Method Applied to Wound Age Estimation: Developing a Stacking Ensemble Model to Improve Predictive Performance Based on Multi-mRNA
Source: Diagnostics (Basel). 2023 Jan 20;13(3):395. doi: 10.3390/diagnostics13030395 (PMC9914838; doi:10.3390/diagnostics13030395)
Supplement: Supplementary file 1 [file diagnostics-13-00395-s001.zip › Supplementary_Material S2.pdf]

Five optimal hyperparameter combinations of machine learning models

Random Forest Classifier: bootstrap=True, ccp_alpha=0.0, class_weight=None, criterion='gini', max_depth=80, max_features='auto', max_leaf_nodes=None, max_samples=None, min_impurity_decrease=0.0, min_impurity_split=None, min_samples_leaf=1, verbose=0, min_samples_split=2, min_weight_fraction_leaf=0.0, n_estimators=200, n_jobs=None, oob_score=False, random_state=0, warm_start=False

Support Vector Machine: C=10, break_ties=False, cache_size=200, class_weight=None, coef0=0.0, decision_function_shape='ovr', degree=5, gamma=1, kernel='rbf', max_iter=-1, probability=True, random_state=3, shrinking=True, tol=0.001, verbose=False

Multilayer Perceptron Classifier: activation='relu', alpha=0.0001, batch_size='auto', beta_1=0.9, beta_2=0.999, early_stopping=False, epsilon=1e-08, hidden_layer_sizes=(64, 128, 256), learning_rate='constant', learning_rate_init=0.001, max_fun=15000, max_iter=200, momentum=0.9,n_iter_no_change=10,nesterovs_momentum=True,power_t=0.5, tol=0.0001, random_state=4, shuffle=True, solver='adam', validation_fraction=0.1, verbose=False, warm_start=False

Gradient Boosting: ccp_alpha=0.0, criterion='friedman_mse', init=None, learning_rate=0.1, loss='deviance',max_depth=5,max_features=None,max_leaf_nodes=None,min_impurity_dec rease=0.0,min_impurity_split=None,min_samples_leaf=1,min_samples_split=2, tol=0.0001, min_weight_fraction_leaf=0.0,n_estimators=50,n_iter_no_change=None,presort='deprecated', random_state=3, subsample=0.5, validation_fraction=0.1, verbose=0, warm_start=False

Stochastic Gradient Descent: alpha=0.0001, average=False, class_weight=None, power_t=0.5, early_stopping=False, epsilon=0.1, eta0=0.0, fit_intercept=True, l1_ratio=0.15, loss='log', learning_rate='optimal', max_iter=50, n_iter_no_change=9, n_jobs=None, warm_start=False penalty='l2', random_state=0, shuffle=True,tol=0.001,validation_fraction=0.1,verbose=0
