# Supplementary material for: Novel Prediction Method Applied to Wound Age Estimation: Developing a Stacking Ensemble Model to Improve Predictive Performance Based on Multi-mRNA
Source: Diagnostics (Basel). 2023 Jan 20;13(3):395. doi: 10.3390/diagnostics13030395 (PMC9914838; doi:10.3390/diagnostics13030395)
Supplement: Supplementary file 1 [file diagnostics-13-00395-s001.zip › Supplementary_Material S3.pdf]

Python Code for models of Training and Validation

#**Import Dependent Packages**

```
from sklearn import datasets
import pandas as pd
import numpy as np
import seaborn as sns
import sklearn.model_selection as ms
import matplotlib.pyplot as plt
from sklearn.preprocessing import StandardScaler
from sklearn.model_selection import StratifiedKFold
from sklearn.model_selection import KFold
from sklearn import metrics
from sklearn.linear_model import LogisticRegression
from sklearn import svm
from sklearn.ensemble import GradientBoostingClassifier
from sklearn.ensemble import RandomForestClassifier
from sklearn.neural_network import MLPClassifier
from sklearn.linear_model import SGDClassifier
from mlxtend.classifier import StackingClassifier
from sklearn.model_selection import train_test_split
from sklearn.multiclass import OneVsRestClassifier
from sklearn.preprocessing import label_binarize
from itertools import cycle
from sklearn import preprocessing
from sklearn import model_selection
from scipy import interp
```

Training for primary model

```
path = 'data.xlsx'
X = pd.read_excel(path,'data(test)').values
Y = pd.read_excel(path,'target(test)').values
```

#Grid search strategy

# Random Forest

```
model = RandomForestClassifier()
params = {'bootstrap': [True,False], 'max_depth': [80, 90, 100, 110], 'n_estimators':
[200,300,400,500], 'random_state' :[0]}
model = ms.GridSearchCV(estimator=model, param_grid=params, cv=5)
model.fit (X_train, y_train)
print (" the optimal parameters:", model.best_params_)
print ("optimal model score:", model.best_score_)
print ("optimal model object:", model.best_estimator_)
for p, s in zip(model.cv_results_['params'], model.cv_results_['mean_test_score']): print (p, s)
```

# Support Vector Machine

```
model = svm(probability=True)
params=[{'kernel':['linear'],'C':[1,10,100,1000]},{'kernel':['poly'],'C':[1,10],'degree':[2,3]},{'kernel'
:['rbf'],'C':[1,10,100,1000], 'gamma':[1,0.1, 0.01, 0.001]}]
model = ms.GridSearchCV(estimator=model, param_grid=params, cv=5)
model.fit(X_train, y_train)
print (" the optimal parameters:", model.best_params_)
print ("optimal model score:", model.best_score_)
print ("optimal model object:", model.best_estimator_)
for p, s in zip(model.cv_results_['params'], model.cv_results_['mean_test_score']):
print (p, s)
```

# Multilayer Perceptron

```
model = MLPClassifier()
params = [{'solver': ['adam'],'hidden_layer_sizes': [(64,128,256)],'random_state': [0]},
{'solver': ['sgd'],'hidden_layer_sizes': [(64,128,256)],'random_state': [0]},
{'solver': ['lbfgs'],'hidden_layer_sizes': [(64,128,256)],'random_state': [0]}]
model = ms.GridSearchCV(estimator=model, param_grid=params, cv=5)
model.fit(X_train, y_train)
print (" the optimal parameters:", model.best_params_)
print ("optimal model score:", model.best_score_)
print ("optimal model object:", model.best_estimator_)
for p, s in zip(model.cv_results_['params'], model.cv_results_['mean_test_score']): print (p, s)
```

# Gradient Boosting

```
model = GradientBoostingClassifier()
params = {'n_estimators': [50,100,150,200], 'subsample': [0.5, 0.6, 0.8], 'max_depth': [3, 5, 6, 7, 10],
'random_state':[0]}
model = ms.GridSearchCV(estimator=model, param_grid=params, cv=5)
model.fit(X_train, y_train)
print (" the optimal parameters:", model.best_params_)
print ("optimal model score:", model.best_score_)
print ("optimal model object:", model.best_estimator_)
for p, s in zip(model.cv_results_['params'], model.cv_results_['mean_test_score']):
print (p, s)
```

# Stochastic Gradient Descent

```
model = SGDClassifier()
params={'max_iter': [50,100,150,200,400],'n_iter_no_change': [1,3,5,7,9],'penalty': ['l2'],
'random_state':[0],'loss':['log'],}
model = ms.GridSearchCV(estimator=model, param_grid=params, cv=5)
model.fit (X_train, y_train)
print (" the optimal parameters:", model.best_params_)
print ("optimal model score:", model.best_score_)
print ("optimal model object:", model.best_estimator_)
for p, s in zip(model.cv_results_['params'], model.cv_results_['mean_test_score']):
print (p, s)
```

# Validation for model

#Internal Validation

# Random Forest

X_train, X_test, y_train, y_test = train_test_split(X, Y, test_size = 0.25, random_state =1,stratify=Y)
clf1= RandomForestClassifier(bootstrap=True, ccp_alpha=0.0, class_weight=None, criterion='gini', max_depth=80, max_features='auto', max_leaf_nodes=None, max_samples=None, n_jobs=None, n_estimators=200, min_impurity_decrease=0.0, min_impurity_split=None, min_samples_leaf=1, verbose=0,min_samples_split=2,min_weight_fraction_leaf=0.0, oob_score=False, random_state=0, warm_start=False)
clf1.fit (X_train, y_train)
prey1 = clf1.predict(X_test)
clf1.score(X_test,y_test)

# Support Vector Machine

svm(C=10, break_ties=False, cache_size=200, class_weight=None, coef0=0.0,degree=5, decision_function_shape='ovr', gamma=1, kernel='rbf', max_iter=-1, probability=True, random_state=3, shrinking=True, tol=0.001, verbose=False)
clf2.fit (X_train, y_train)

# Multilayer Perceptron

MLPClassifier(activation='relu', alpha=0.0001, batch_size='auto', beta_1=0.9, beta_2=0.999, early_stopping=False, epsilon=1e-08, hidden_layer_sizes=(64, 128, 256), learning_rate='constant', learning_rate_init=0.001, max_fun=15000, max_iter=200, momentum=0.9, n_iter_no_change=10, nesterovs_momentum=True, power_t=0.5, random_state=4, shuffle=True, solver='adam', tol=0.0001, validation_fraction=0.1, verbose=False, warm_start=False)
clf3.fit (X_train, y_train)

# Gradient Boosting

GradientBoostingClassifier(ccp_alpha=0.0, criterion='friedman_mse', init=None, learning_rate=0.1, loss='deviance', max_depth=5, max_features=None, max_leaf_nodes=None, min_impurity_decrease=0.0,min_impurity_split=None,min_samples_leaf=1, min_samples_split=2, min_weight_fraction_leaf=0.0, n_estimators=50, n_iter_no_change=None, presort='deprecated', random_state=3,subsample=0.5,tol=0.0001, validation_fraction=0.1, verbose=0, warm_start=False)
clf4.fit (X_train, y_train)

# Stochastic Gradient Descent

SGDClassifier(alpha=0.0001, average=False, class_weight=None, early_stopping=False, epsilon=0.1, eta0=0.0, fit_intercept=True, l1_ratio=0.15, learning_rate='optimal', loss='log', max_iter=50, n_iter_no_change=9, n_jobs=None, penalty='l2', power_t=0.5, random_state=0, shuffle=True, tol=0.001, validation_fraction=0.1, verbose=0, warm_start=False)
clf5.fit (X_train, y_train)

# Stacking Ensemble Learning (26)

lr = LogisticRegression()
sclfA= StackingClassifier(classifiers=[clf1, clf2],# [clf1,or clf2, or clf3, or clf4, or clf5]
use_probas=True,average_probas=False, meta_classifier=lr)
print ('3-fold cross validation:\n')
for clf, label in zip ([clf1, clf2, sclfA], ['RF', 'svm', 'StackingClassifier']): scores= model_selection.cross_val_score(clf, X_train, y_train, cv=5, scoring='accuracy')

```
print ("Accuracy: %0.2f (+/- %0.2f) [%s]" % (scores.mean(), scores.std(), label))
sclfA=StackingClassifier(classifiers=[clf1,clf2],use_probas=True,average_probas=False,
meta_classifier=lr)
sclfA.fit(X_train, y_train)
preyA= sclfA.predict(X_test)
sclfA.score(X_test,y_test)
```

#External Validation

#**Data Iuput**

```
X1 = pd.read_excel(path,'data（validation）').values
Y1 = pd.read_excel(path,'target（validation）').values
X_train, X_test, y_train, y_test = train_test_split(X, Y, test_size = 0.25, random_state =1,stratify=Y)
```

# Multilayer Perceptron

```
MLPClassifier(activation='relu', alpha=0.0001, batch_size='auto', beta_1=0.9, beta_2=0.999,
early_stopping=False, epsilon=1e-08, hidden_layer_sizes=(64, 128, 256), learning_rate='constant',
learning_rate_init=0.001, max_fun=15000, max_iter=200, momentum=0.9, n_iter_no_change=10,
nesterovs_momentum=True, power_t=0.5, random_state=4, shuffle=True, solver='adam',
tol=0.0001, validation_fraction=0.1, verbose=False, warm_start=False)
clf3.fit (X_train, y_train)
```

# Stacking Ensemble Learning

```
clf1= RandomForestClassifier(bootstrap=True, ccp_alpha=0.0, class_weight=None, criterion='gini',
max_depth=80,max_features='auto',max_leaf_nodes=None,max_samples=None,verbose=0,
min_impurity_decrease=0.0,min_impurity_split=None,min_samples_leaf=1,n_jobs=None,
min_samples_split=2, min_weight_fraction_leaf=0.0, n_estimators=200, oob_score=False,
random_state=0, warm_start=False)
clf1.fit(X_train, y_train)
prey1 = clf1.predict(X_test)
clf1.score(X_test,y_test)
clf2=SVC(C=10, break_ties=False, cache_size=200, class_weight=None, coef0=0.0,
decision_function_shape='ovr', degree=5, gamma=1, kernel='rbf', max_iter=-1, probability=True,
random_state=3, shrinking=True, tol=0.001, verbose=False)
clf2.fit (X_train, y_train)
prey = clf2.predict(X_test)
clf2.score(X_test,y_test)
clf3 = MLPClassifier(activation='relu', alpha=0.0001, batch_size='auto', beta_1=0.9, beta_2=0.999,
early_stopping=False, epsilon=1e-08, hidden_layer_sizes=(64, 128, 256), learning_rate='constant',
learning_rate_init=0.001, max_fun=15000, max_iter=200, momentum=0.9, n_iter_no_change=10,
nesterovs_momentum=True, power_t=0.5, random_state=4, shuffle=True, solver='adam',
tol=0.0001, validation_fraction=0.1, verbose=False, warm_start=False)
clf3.fit (X_train, y_train)
prey = clf3.predict(X_test)
clf3.score(X_test,y_test)
lr = LogisticRegression()
sclf = StackingClassifier(classifiers=[clf1, clf2, clf3],use_probas=True, average_probas=False,
```

```
meta_classifier=lr)
print ('3-fold cross validation:\n')
for clf, label in zip ([clf1, clf2, clf3, sclf], ['RF', 'svm', 'mplc', 'StackingClassifier']):
scores = model_selection.cross_val_score(clf, X_train, y_train, cv=5, scoring='accuracy')
print ("Accuracy: %0.2f (+/- %0.2f) [%s]" % (scores.mean(), scores.std(), label))
sclf = StackingClassifier(classifiers=[clf1, clf2, clf3],use_probas=True,average_probas=False,
meta_classifier=lr)
sclf.fit (X_train, y_train) preyc = sclf.predict(X1)
```
